# Quantitative and Selective Analysis of Feline Growth Related Proteins Using Parallel Reaction Monitoring High Resolution Mass Spectrometry

**DOI:** 10.1371/journal.pone.0167138

**Published:** 2016-12-01

**Authors:** Mårten Sundberg, Emma M. Strage, Jonas Bergquist, Bodil S. Holst, Margareta Ramström

**Affiliations:** 1 Analytical Chemistry, Department of Chemistry–BMC and Science for Life Laboratory, Uppsala University, Uppsala, Sweden; 2 Department of Clinical Sciences, Swedish University of Agricultural Sciences, Uppsala, Sweden; 3 Clinical Pathology Laboratory, University Animal Hospital, Swedish University of Agricultural Sciences, Uppsala, Sweden; Leibniz-Institut fur Analytische Wissenschaften - ISAS eV, GERMANY

## Abstract

Today immunoassays are widely used in veterinary medicine, but lack of species specific assays often necessitates the use of assays developed for human applications. Mass spectrometry (MS) is an attractive alternative due to high specificity and versatility, allowing for species-independent analysis. Targeted MS-based quantification methods are valuable complements to large scale shotgun analysis. A method referred to as parallel reaction monitoring (PRM), implemented on Orbitrap MS, has lately been presented as an excellent alternative to more traditional selected reaction monitoring/multiple reaction monitoring (SRM/MRM) methods. The insulin-like growth factor (IGF)-system is not well described in the cat but there are indications of important differences between cats and humans. In feline medicine IGF–I is mainly analyzed for diagnosis of growth hormone disorders but also for research, while the other proteins in the IGF-system are not routinely analyzed within clinical practice. Here, a PRM method for quantification of IGF–I, IGF–II, IGF binding protein (BP) –3 and IGFBP–5 in feline serum is presented. Selective quantification was supported by the use of a newly launched internal standard named QPrEST™. Homology searches demonstrated the possibility to use this standard of human origin for quantification of the targeted feline proteins. Excellent quantitative sensitivity at the attomol/μL (pM) level and selectivity were obtained. As the presented approach is very generic we show that high resolution mass spectrometry in combination with PRM and QPrEST™ internal standards is a versatile tool for protein quantitation across multispecies.

## Introduction

Insulin-like growth factor–I and–II (IGF–I and–II) are important regulators of growth and metabolism in both humans and animals. In adult humans almost all IGFs are bound to insulin-like growth factor binding proteins (IGFBP 1–6) which in addition to modulating IGF-bioavailability exert their own biological effects [1,2]. In adult humans, IGFs circulate mainly as ternary complexes bound to IGFBP–3 or IGFBP–5 and the acid labile subunit (IGFALS). The ternary complex prolongs the half-life of IGFs and thus is a major determinant of IGF-concentrations [2]. The IGF-system is not well described in the cat, but there are indications of important differences between cats and humans [3]. To understand the IGF–system in the cat, and to improve clinical diagnoses, it would be beneficial to be able to measure IGF–I,–II and IGFBPs. In feline medicine IGF–I is mainly analyzed for diagnosis of growth hormone disorders but also for research [4]. IGF–II, IGFBP–3 and –5 are not routinely analyzed in clinical practice and there are to our knowledge no commercial validated assays for cats. Large variability for human IGF-I immunoassays has been reported [5]. The College of American Pathologists (CAP) proficiency testing program for IGF–I demonstrated an interlaboratory variability of up to 34% CV from September 2011 to March 2013, while a mass spectrometry-based method demonstrated better reproducibility (CV<16%) [6]. One study demonstrated large variability when measuring feline IGF-I with four non cat-specific immunoassays [7]. Applying human immunoassays for analysis of samples from other species might cause problems including no or weak reactivity or unwanted cross-reactivity, and the assays therefore need to be thoroughly validated before use. Measurements of IGFs with immunoassays are difficult due to interference of IGFBPs, which may cause both false high and false low values depending on assay [8].

A targeted mass spectrometry-based method would be an attractive alternative to immunoassays. Such an approach is not restricted to selected species and the time-consuming and expensive work to produce an affinity reagent is circumvented. The concept is targeted selection of a peptide (precursor) and quantification based on 3–6 fragments of that peptide. Targeted MS is usually performed on a triple quadrupole and is referred to as selected reaction monitoring/multiple reaction monitoring (SRM/MRM) [9]. The time-consuming and difficult part of SRM/MRM method development is to find a good precursor/product ion pair [10]. Lately, an alternative approach, referred to as parallel reaction monitoring (PRM) [11–13], has been developed for quadrupole-orbitrap mass spectrometers. In a PRM analysis, all transitions of the targeted precursors are measured and the selection of fragments for quantification is done post-acquisition. This in combination with high selectivity in the quadrupole MS and high-resolution in the Orbitrap MS makes it a very good method for targeted proteomics in complex matrices such as serum or plasma [14]. A commonly used strategy for absolute quantification with SRM/MRM experiments is to include synthetic heavy isotopically labelled reference peptides. In this study, a recently launched product named QPrEST™ was used as internal standard. QPrESTs™ are 50–150 amino acid long sequence segments of human proteins with heavy isotope-labelled (^15^N, ^13^C) Lysine and Arginine, developed within the Human Protein Atlas project [15–17]. QPrESTs™ are digested together with the sample giving the potential advantage that incomplete or unspecific digestion does not corrupt the results, which can be an issue using synthetic isotope-labelled peptides. The most similar reagents on the market are the so-called QconCAT constructs [18,19]. QconCATs are artificial proteins, consisting of tryptic peptides from several proteins in sequence. There are variants of QconCAT available, including reagents with natural flanking sequences [20]. QPrESTs™ are produced from an already existing library of well validated constructs, covering more than 80% of the human protein-coding genes [17].

For humans, several quantitative mass spectrometry-based methods have been set up for determination of IGF–I, –II and IGFBP–3. There are publications of both SRM/MRM [6,21–25] and top-down [26–28] approaches. Recently it has also been shown that a mass spectrometry-based method decreases the interlaboratory variance [6] compared to affinity based methods. As regards to other species, mass spectrometry-based quantification studies have been published on IGF-I in horse plasma/serum [29,30]. To the best of the authors’ knowledge no mass spectrometry-based quantification study has been published on feline IGF–I, –II or any IGFBP. The aim of this study was therefore to set up a quantitative targeted mass spectrometry-method for IGF–I, –II, IGFBP–3 and IGFBP–5 in feline sera. We demonstrate that the PRM-based strategy enables accurate quantification of the four target proteins. In addition, QPrESTs™, originally developed to represent human proteins, are shown to be useful internal standards also in applications concerning feline proteins.

## Materials and Methods

### Chemicals and reagents

Acetonitrile (ACN), acetone, formic acid (FA) were purchased from Merck (Darmstadt, Germany). Ammonium bicarbonate (NH_4_HCO_3_), urea, dithiothreitol (DTT) and iodoacetamide (IAA) were obtained from Sigma-Aldrich (St. Louis, MO, USA). For the tryptic digestion, trypsin (Sequencing grade modified, Promega, Madison, WI, USA) was used. Ultrapure water was prepared by Milli-Q water purification system (Millipore, Bedford, MA, USA).

### Feline serum samples and gel fractionated feline serum

Approval for blood sampling of the cats was given by the Swedish Ethical Committee (C301/10) and the Swedish Board of Agriculture (31-10551/10). All blood samples were centrifuged after 30–60 minutes in room temperature and serum frozen at -80°C until use. Serum from one cat was separated with size exclusion chromatography on a Superdex 75 column (17-5174-01, GE Healthcare, Little Chalfont, U.K.) in acidic conditions to separate IGFs from IGFBPs. A protocol published by Mohan and Baylink was followed, with the exception that samples were incubated with 1 M acetic acid containing 0.1 M NaCl for 30 minutes before loaded onto the column [31]. In short, 0.1 mL serum was diluted with 0.4 mL of the acetic acid/NaCl solution, incubated for 30 minutes at room temperature and filtered to remove particles (Whatman, Rezist, 0.2 μm, GE Healthcare). A volume of 0.4 mL of the sample was loaded onto the column and eluted at 0.5 mL/min. Fractions were collected every second minute and dried in a SpeedVac system. A commercially available IGF-I ELISA (Mediagnost, Reutlingen) or an in house IGF-II assay was used for the immunoreactivity measurements in each fraction. Three of the gel fractions (Fraction I, II and III) were selected for analysis with mass spectrometry. In total seven feline sera samples were included in the study (A-G). The samples were chosen to represent cats with potentially different levels of the target proteins. Three of the samples were collected from healthy cats (A-C). Three of the samples were from cats with diabetes mellitus (D-F), where Sample F was collected after insulin treatment of cat E. Finally, a sample from a cat with diabetes mellitus diagnosed with acromegaly was included (Sample G). Sample A-G were not fractionated.

### Protein standards

**Fig 1:**
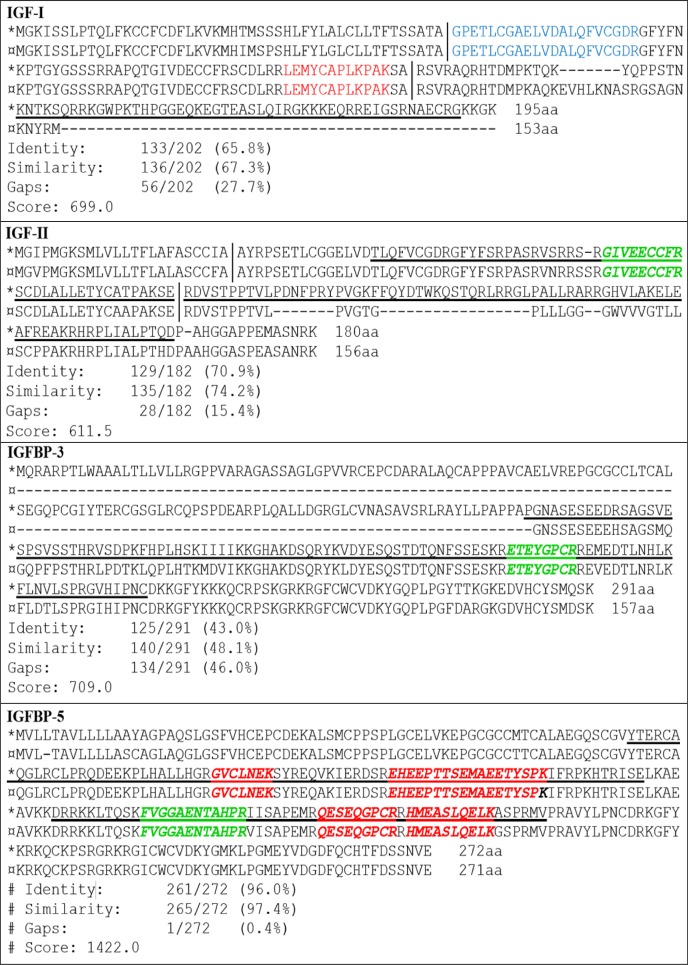
Homology study of the four selected cat proteins and their human analogues. Human (*) and feline (¤) amino acid sequence with QPrEST™ sequence underscored in the human sequence. IGFBP–5 has two available QPrESTs™ while IGF–II and IGFBP–3 have one each. Tryptic peptides in QPrESTs™ matching peptides found in the feline sequence are market in bold italic (green peptides were used for quantification and the red ones were evaluated but not used). For IGF-I there was no tryptic peptide in the QPrEST™ that matched any feline peptide. The blue peptide is the synthetic NEPTune™ peptide used for quantification and the red peptide was evaluated but not used. The shorter mature protein is marked between ││ for IGF–I and IGF–II. Similarity scores from EMBOSS Needle is presented below the sequences.

To be able to match tryptic peptides in QPrESTs™ with feline sequences, EMBOSS Needle [32] was used for sequence alignment of human and feline amino acid (aa) sequences, see Fig 1. QPrEST™ containing peptides matching with feline IGF–II (QPrEST22489), IGFBP–3 (QPrEST23429) and IGFBP–5 (QPrEST23781 and QPrEST23782), ≥99% isotopic purity and ≥80% peptide purity, were obtained from Atlas antibodies (Stockholm, Sweden). For IGF–I two peptides (GPETLCGAELVDALQFVCGDR and LEMYCAPLKPAK) synthesized with heavy labeled (^15^N, ^13^C) Lysine and Arginine (≥99% isotopic purity and ≥95% peptide purity) were purchased from New England Peptides, referred to as NEPTune™ peptides (Gardner, MA, USA). Human recombinant IGF–I and IGF–II proteins were purchased from Immunological & Biochemical Testsystems (Binzwangen, Germany). A human control serum with specified concentration of IGF-I, Control KS2 validated for Mediagnost IGF-I E20 ELISA, was purchased from Mediagnost (Reutlingen, Germany).

### In-solution tryptic digestion

The protein content in feline sera was measured with the Bradford protein assay (Bio-Rad Laboratories, Hercules, CA, USA). Aliquots corresponding to approximately 35 μg total protein were taken for in-solution digestion. The volume was adjusted with 0.4 M NH_4_HCO_3_, pH 8 to a total volume of 200 μL. QPrESTs™ for IGFBP-3 and -5 were spiked to final concentrations of 2.0 and 1.0 fmol/μL, respectively. The QPrEST™ for IGF–II was spiked to 3.0 fmol/μL. The samples were sonicated for 1 minute and after that 10 μL of 45 mM DTT was added and the sample was kept at 50°C for 15 min to reduce the proteins. To irreversibly carbamidomethylate the cysteines, 10 μL of 100 mM IAA was added, followed by 15 min incubation at room temperature in darkness. For the digestion, trypsin (~5% w/w) was added and the sample was incubated over night at 37°C. The heavy labeled synthetic peptides were spiked in to a final concentration of 0.59 fmol/μL. Each of the tryptically digested samples was divided in 4 aliquots corresponding to ~8.75 μg protein and the aliquots were desalted on a ZipTip® C18 column (Merck Millipore). The aliquots were dried in a SpeedVac system and were then re-dissolved in 20 μL of 0.1% FA. The tip was activated by 3 x 10 μL of 100% ACN and equilibrated with 3 x 10 μL of 0.1% FA. After this the sample was coupled to the matrix by 5 repeated cycles of 10 μL sample loading. The tip was then washed with 5 x 10 μL 0.1% FA. Finally the sample was eluted in 20 μL 80% ACN, 0.1% FA by 5 cycles of aspirating and dispensing. After desalting, the sample was dried in a SpeedVac system. Before analysis by nanoLC-LTQ-Orbitrap mass spectrometry the peptides from each aliquot were re-dissolved in 20 μL of 0.1% FA in Milli-Q water. The protocol was adjusted for the gel fractionated feline serum sample, the amount of DTT and IAA was reduced to 1 μL and the ZipTip® C18 column step was excluded since pre-purification of the sample was obtained in the size exclusion chromatography step. The human control serum was prepared twice in exactly the same way as the feline sera samples, by two different analysts.

### Shotgun nanoLC-LTQ-Orbitrap-MS/MS analysis

Online nano-LC separations were obtained with an EASY-nLC II system (ThermoFisher). A volume of 5 μL sample was loaded onto a pre-column (EASY-Column, 2 cm, inner diameter 100 μm, 5 μm, C18-A1, ThermoFisher Scientific) at a maximum pressure of 280 bar. The peptides were then eluted onto an EASY-column, 10 cm, inner diameter 75 μm, 3 μm, C18-A2 (ThermoFisher Scientific), which was used for the separation. A flow rate of 200 nL/min using mobile phase A (Milli-Q water with 0.1% FA) and B (ACN with 0.1% FA) was set for the separation. A 90 min 2-step gradient, 2% B up to 50% B in 75 min, followed by a 15 min wash step of 100% B, was used. The EASY-nLC II system was connected via a nano-flex ion source to a LTQ Orbitrap Velos Pro ETD mass spectrometer (ThermoFisher Scientific). The spray voltage was set to 2.0 kV. The instrument was operated in data dependent mode to automatically switch between high resolution mass spectrum and low resolution in the LTQ. The system was controlled through LTQ Tune PlusTune 2.7 and Xcalibur 2.1. A survey scan was performed from m/z 400 to 2000 at a resolution of 100,000 (at *m/z* 400) and the 10 most abundant ion peaks were CID fragmented for each full scan cycle. Screening was done for charge state +2, +3 and +4 and the dynamic exclusion was set to 30 seconds. The mass window for precursor ion selection was set to 1.9 m/z. For the MS/MS, a normalized collision energy of 35%, activation time of 10 ms and activation q of 0.25 were used. The fragments were scanned in the low pressure cell of the ion trap and detected with a secondary electron multiplier.

### NanoLC-Q Exactive Plus-PRM analysis

The PRM analyses were performed on a Q Exactive Plus Orbitrap mass spectrometer (ThermoFisher Scientific). An EASY-nLC 1000 system (ThermoFisher Scientific) was used for the peptide separation. The columns and the ion source were analogous to what was used with EASY-nLC II system in the shotgun set up. A flow rate of 250 nL/min using mobile phase A (Milli-Q water with 0.1% FA) and B (ACN with 0.1% FA) was used for the separation. Four μL of the tryptic digested feline serum samples were injected. A 60 min 3-step gradient, 4% B up to 40% B in 40 min, 40% B -75% B in 10 min followed by a 10 min wash step of 100% B, was used. The system was controlled through Q Exactive Plus Tune 2.5 and Xcalibur 3.0.

The PRM method combined two scan events starting with a full scan event followed by targeted MS/MS for the doubly and/or triply charged precursor ions scheduled in an inclusion list with a ±2 min retention time window. The full scan event employed a m/z 300–800 mass selection, an Orbitrap resolution of 140,000 at m/z 200, an automatic gain control (AGC) target value of 3*10^6^, and maximum fill times of 250 ms. The targeted MS/MS was run at an Orbitrap resolution of 35,000 at m/z 200, an AGC target value of 1*10^6^, and maximum fill times of 200 ms. The targeted peptide was isolated using a 1.2 m/z unit window. Fragmentation was performed with normalized collision energy (NCE) of 27 eV. The most repeatable charge states regarding signal intensity were chosen for the selected peptides. In total nine peptides and their corresponding heavy peptides were comprised in the inclusion list. For some of them the differences between the two charge states were not obvious and both were kept for the analysis of feline sera (Table 1).

**Table 1:** Targeted peptides included in the PRM method. Bold,underscored peptides were used for final quantification. C-terminal arginine (R) and Lysine (K) were heavy labeled. All cysteines are carbamidomethylated. The fragments selected for quantification are given, and all these fragments were singly charged.

**IGF I**					
**[m/z] (Th)**	**Charge**	**Sequence**	**Position**	**RT (min)**	**Fragments**
**769.6963**	3+	**GPETLCGAELVDALQFVCGDR (light)**	1–21	41.5	y_4_, y_5_, y_6_, y_7_, y_10_, b_9_
773.0324	3+	GPETLCGAELVDALQFVCGDR (heavy)	1–21	41.5	
710.8700	2+	LEMYCAPLKPAK (light)	57–68	23.8	
714.8771	2+	LEMYCAPLKPAK (heavy)	57–68	23.8	
474.2491	3+	LEMYCAPLKPAK (light)	57–68	23.8	
476.9205	3+	LEMYCAPLKPAK (heavy)	57–68	23.8	
479.5807	3+	LEM[+16.0]YCAPLKPAK (light)	57–68	20.6	
482.2521	3+	LEM[+16.0]YCAPLKPAK (heavy)	57–68	20.6	
**IGF II**					
**[m/z] (Th)**	**Charge**	**Sequence**		**RT (min)**	
**585.2575**	2+	**GIVEECCFR (light)**	41–49	22.8	y_3,_ y_4_, y_5_,y_6_, y_7,_ b_3_
590.2617	2+	GIVEECCFR (heavy)	41–49	22.8	
**IGFBP-3**				**RT (min)**	
**[m/z] (Th)**	**Charge**	**Sequence**			
**506.2136**	2+	**ETEYGPCR (light)**	73–80	15.0	y_2,_ y_4_, y_5_
511.2178	2+	ETEYGPCR (heavy)	73–80	15.0	
**IGFBP-5**					
**[m/z] (Th)**	**Charge**	**Sequence**		**RT (min)**	
410.2051	2+	GVCLNEK (light)	97–103	15.7	
414.2122	2+	GVCLNEK (heavy)	97–103	15.7	
698.9651	3+	EHEEPTTSEMAEETYSPK (light)	117–134	21.4	
701.6365	3+	EHEEPTTSEMAEETYSPK (heavy)	117–134	21.4	
628.3125	2+	FVGGAENTAHPR (light)	164–175	15.1	
633.3167	2+	FVGGAENTAHPR (heavy)	164–175	15.1	
**419.2108**	3+	**FVGGAENTAHPR (light)**	164–175	15.1	y_2,_ y_7_, y_10_
422.5469	3+	FVGGAENTAHPR (heavy)	164–175	15.1	
545.7327	2+	QESEQGPCR (light)	184–192	10.3	
550.7369	2+	QESEQGPCR (heavy)	184–192	10.3	
593.3003	2+	HMEASLQELK (light)	194–203	21.7	
597.3074	2+	HMEASLQELK (heavy)	194–203	21.7	
395.8693	3+	HMEASLQELK (light)	194–203	21.7	
398.5407	3+	HMEASLQELK (heavy)	194–203	21.7	

### Data analysis

Protein identification of the shotgun runs was performed using Proteome Discoverer, version 1.4.1.14 (ThermoFischer Scientific). Searches were performed using Sequest HT including the Percolator [33]. The searches were done against a feline reference proteome without isoforms (taxonomy 9685), downloaded from www.uniprot.org. The parameters for the search were set to: fixed modifications: Carbamidomethyl (C), variable modifications: Deamidated (N, Q) and Oxidation (M), precursor mass tolerance: 10 ppm, fragment mass tolerance: 0.6 Da and maximum two missed cleavage sites. The S/N threshold was set to 1.5. The search results were validated using the Percolator and a 5% false discovery rate (FDR) was used. A minimum of two unique peptides per protein were used for identification. For the data analysis and quantification of the PRM runs, the Skyline software [34] was used. The quantification was based on the sum of the area under the curves (AUC) of three to six fragments of the selected peptides and the ratio between sample peptide fragments and the heavy isotope peptide fragments. Each sample was measured three times and the mean and standard deviation of the determined concentrations were calculated.

### Determination of linearity, limit of detection and limit of quantification

QPrESTs™ and the longer NEPTune™ peptide were used in a spike-in experiment to determine the linearity, limit of detection (LOD) and limit of quantification (LOQ) of the target proteins, applying the optimized PRM-method. The QPrESTs™ were digested and thereafter spiked into digested cat sera to final concentrations of 0.05, 0.25, 0.5, 1.0, 2.5 and 12.5 fmol/μL. The NEPTune™ peptides were spiked into the same samples without prior treatment, to final concentrations of 0.03, 0.15, 0.29, 0.59, 1.5 and 7.3 fmol/μL, respectively. The samples were analyzed in three replicate runs. LOD and LOQ were calculated based on linear regression and the following formulas [35]: LOD = 3S_a_/b, LOQ = 10S_a_/b, where S_a_ is the standard deviation of the y-intercepts and b is the slope of the standard dilution curve. The SkyLine software was applied to extract AUC of the fragments and the built in tool Data Analysis in Microsoft Excel 2010 was used to perform linear regression and statistical evaluation.

## Results

### Sample screening and internal standard selection

The aim of this study was to set up a targeted mass spectrometry method for quantification of IGF–I, IGF–II, IGFBP–3 and IGFBP–5 in feline sera. Since no feline protein standards were available, gel fractionation was performed to concentrate the target proteins and remove interfering abundant proteins. All the targeted proteins were classified as “uncharacterized protein” in the UniProt database. It was therefore important to validate their sequences prior to peptide selection. With guidance of results from ELISA runs targeting IGF–I and –II, three gel fractions were selected for mass spectrometry analysis. Shotgun analysis on an LTQ Orbitrap Velos Pro ETD mass spectrometer resulted in a number of detected proteins, and the four target proteins were among the identified ones, see Table 2 and Supporting Information S1A–S1C Table. The detected sequence coverage was 90, 50, 29 and 14% for IGF-I, IGF-II, IGFBP–3 and –5, respectively. Thus, the results confirmed parts of the uncharacterized sequences and showed that there were a number of peptides that could potentially be used for quantification.

**Table 2:** Result of shotgun analyses of gel fractionated cat sera samples.

	Fraction I	Fraction II	Fraction III
**Total number of detected proteins**	75	50	27
**IGF-I**	Present	Present	Present
**IGF-II**	Not present	Present	Present
**IGFBP-3**	Present	Not present	Not present
**IGFBP-5**	Present	Not present	Not present

An additional objective of the study was to explore if human-derived QPrEST™s could be used as internal standards for quantification of cat proteins. Alignment of human (canonical sequence used) and feline sequences revealed that the sequences of the mature form of IGF–I (70 aa) is identical to the human sequence and the mature form of IGF–II (68 aa) differs in only 3 aa from the human sequence. If the first gap in the feline IGFBP–3 sequence is not considered, there is 89% similarity between feline and human proteins. Moreover, it was demonstrated that three of the four target proteins had matching tryptic peptides with at least one QPrEST™ (Fig 1). For IGF–II and IGFBP-3, one feline tryptic peptide per protein matched with the sequence, while five matching tryptic peptides originating from two QPrEST™s were identified for IGFBP–5. It was therefore concluded that QPrEST™s could be used as internal standards for quantification of these three proteins. For IGF–I there was no matching QPrEST™ available even though the mature human and feline IGF–I proteins are identical. Therefore other types of internal standards were required for quantification of IGF-I, and synthetic heavy isotope-labeled peptides (NEPTune™) were selected.

### Method optimization

Given the sequence similarity, recombinant human IGF–I and IGF–II proteins were used for method optimization of the PRM-method on the Q Exactive Plus Orbitrap MS. A 60 minute LC-gradient was applied to ensure good separation of the tryptic peptides. Several parameters including AGC, maximum injection time, resolution, isolation window and NCE were optimized. The final settings for the method can be found in the experimental section. An inclusion list with doubly and triply charged peptides, based on tryptic QPrEST™ peptides and the NEPTune™ peptides, was set up and included in the PRM-method (Table 1). After careful evaluation, one peptide per protein was selected for quantification, see Table 1. Peptide IGF–I (1–21) 3+, provided six MS/MS-fragments that could be used for quantification, with an overall better performance than IGF–I (57–68) 2+. It was observed that methionine oxidation occurred for peptide IGF–I (57–68) 2+ (Table 1), resulting in differences between runs. The IGF–II (41–49) 2+, peptide also produced six reproducible fragments. The IGFBP–3 (73–80) 2+, peptide produced one dominating fragment, but in total there were three fragments used for quantification. IGFBP–5 was the most challenging protein to quantify, since it was of lowest concentration. The peptide IGFBP–5 (164–175) 3+, was selected for quantification based on spectrum reproducibility and signal intensities.

**Fig 2:**
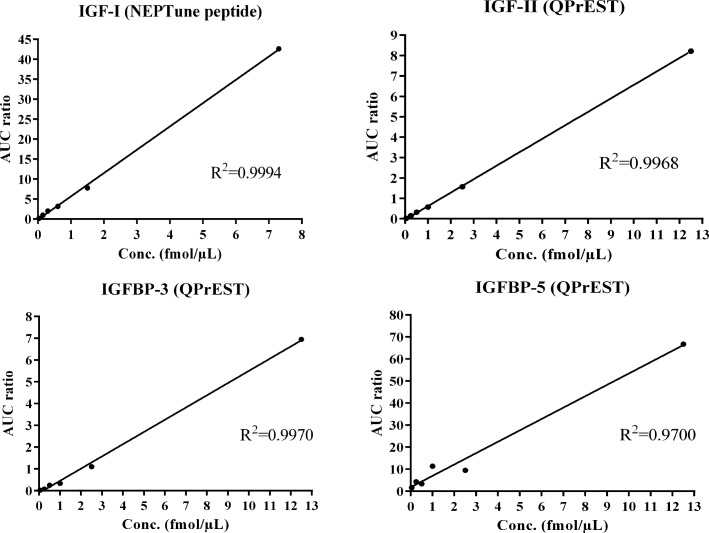
Internal standard dilution series. QPrEST™ and NEPTune™ peptides spiked into cat serum, analyzed with the PRM method at different concentrations. The ratio synthetic/native peptide is plotted against the spiked concentration.

With the optimized method, evaluation of the linearity, limit of detection (LOD) and limit of quantification (LOQ) in a spike-in experiment, into tryptic digested cat serum, was performed. The heavy labeled peptides, i.e. the QPrEST™s and NEPTune™ peptides, were added in known amounts and normalized towards the corresponding native peptides. Linear regression of the normalized signals of the four internal standard peptides resulted in correlation coefficients of 0.9994, 0.9968, 0.9970 and 0.9700 for IGF-I, IGF-II, IGFBP-3 and IGFBP-5, respectively (Fig 2). LOD and LOQ at the attomol/μL (pM) level were established in the sample matrix, i.e. tryptic digest of cat serum, see Table 3. The detection limits were also recalculated to corresponding concentrations in native serum, assuming that the tryptic digestion of the proteins in the sample matrix is complete. The calculations are shown in Supporting Information, S1 File.

**Table 3:** LOD and LOQ values for the quantified peptides in the diluted feline serum and corresponding values for native serum.

	IGF–I		IGF–II		IGFBP–3		IGFBP–5	
	Diluted serum	Native serum	Diluted serum	Native serum	Diluted serum	Native serum	Diluted serum	Native serum
LOD (fmol/μL)	0.043	7.8	0.068	12	0.15	28	0.15	28
LOQ (fmol/μL)	0.14	26	0.23	41	0.51	92	0.51	93

### Quantification in feline sera

**Fig 3:**
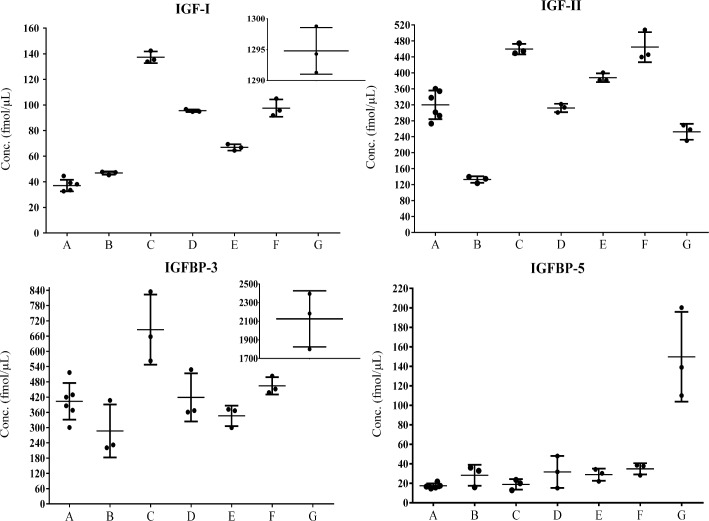
Quantified concentrations of the four targeted proteins in seven samples of feline sera. The average concentrations and standard deviation are given (n = 3 for Samples B-G, n = 6 for Sample A). Samples A-C are samples from healthy cats, D and E were collected from cats with diabetes mellitus before insulin treatment, Sample F was collected from the same cat as Sample E after insulin treatment. Sample G was collected from a cat with acromegaly.

Finally, the concentrations of IGF–I, IGF–II, IGFBP–3 and IGFBP–5 were determined in feline serum samples. To get an indication of the repeatability of the method, one of the samples was analyzed in three replicates from two different preparations. These six measurements gave acceptable relative standard deviation values of 8, 11, 15 and 14% for IGF–I, IGF–II, IGFBP–3 and IGFBP–5, respectively. The concentrations of the four target proteins, determined in three replicate runs, are shown for seven cat sera samples in Fig 3. Raw data and calculations are given in Supporting Information S2 File. It was observed that IGF-II concentrations were higher than IGF-I concentrations in the non-acromegalic cats. Serum from the cat suffering from acromegaly contained significantly higher levels of IGF-I, IGFBP-3 and IGFBP-5 than serum from healthy and from diabetic cats. IGF-II was not elevated in this cat.

## Discussion

In this study, a PRM-method was set up for quantification of four feline growth related hormones (IGF–I,–II, IGFBP–3 and –5). There are no previously established methods to measure feline IGF-II, IGFBP–3 or IGFBP–5. To our knowledge, the present method is the first one developed for quantifying all four proteins in cat serum. One advantage of the MS-based method over immunoassays is that all target proteins are measured within the same experimental run. This reduces both variation due to methodology and the amount of serum needed for analysis, which can be a great advantage in cats and other small animals.

All four targeted proteins were classified as “uncharacterized protein” in the UniProt database. However, a shotgun analysis confirmed parts of their sequences and revealed potential target peptides. This study is the first one to investigate the applicability of QPrESTs™ for quantification of proteins in a non-human sample, feline serum. An advantage of using QPrESTs^TM^ and other protein standards, including QconCATs, is that they are digested together with the target proteins, compensating for incomplete cleavage. In addition, the proteins are expressed in *E*.*Coli*, and are therefore relatively cheap to produce and purchase. After digestion, several peptides are available for quantification. Even though QPrESTs™ are of human origin, high similarity between cat and human target proteins was confirmed (Fig 1), supporting the use of these reagents in feline studies. Repeatable results were obtained for the three target proteins quantified using QPrESTs™. However, a limitation when quantifying non-human proteins is, as demonstrated in this study, that there are in general fewer tryptic peptides that can be selected for quantification. Solely one tryptic fragment was available for two of the proteins, IGF-II and IGFBP-3. QPrESTs™ were still considered good options since they are produced from an already existing library of well validated constructs and built from sequences known to be of low sequence similarity with other proteins in the human proteome. Studies have demonstrated that natural flanking sequences improve the performance of both QconCAT and synthetic peptides [20]. All tryptic peptides derived from QPrESTs are flanked by the native human sequence, and the feline and human sequences are similar in proximity to the cleavage sites (Fig 1.), which should be an advantage. Many validated MS-based methods rely on quantification of one single tryptic peptide [36]. Surprisingly, no matching QPrEST™ was found for IGF-I, despite the fact that the mature human and feline IGF-I proteins are identical. The explanation for that is that the QPrEST™ was produced towards a sequence of the E-domain of pro-IGF-I, which is not a part of the mature IGF-I.

The samples in this study were selected with the aim to analyze serum from healthy cats as well as from cats for which IGF-I is routinely measured in clinical practice, i.e., diabetic and acromegalic cats. Protein concentrations could be determined for all categories of samples. The PRM method delivered detection limits on the attomol/μL (pM) level in the sample matrix. Analysis of IGF–I, IGF–II and IGFBP–3 gave concentrations above the calculated LOQ in all investigated cat serum samples. The lowest concentrations were determined for IGFBP-5. Five of seven samples were above the LOD for this protein, but only the sample from the acromegalic cat, Sample G, gave a value above the calculated LOQ. Thus, the exact quantity of this protein could not be determined, but a significantly higher concentration was measured in serum from the acromegalic cat. The peptide used for quantification of IGFBP–5 eluted early in the gradient and might therefore in this set-up be affected by the void volume.

There is no gold standard method for analysis of IGF–I in cats and no reference material available. Hence it is difficult to state true concentrations. To further explore the accuracy of the PRM-approach, a human control serum used for an IGF-I ELISA, with a specified IGF-I concentration of 396–596 ng/mL, was analyzed. Since the present method was optimized against an IGF-I-peptide common to the human and cat protein, the method should be well applicable for analysis of human samples. The analysis resulted in values within the given range (Supporting Information S3 File). Recently, an approach based on cleavable reporter peptides to recalibrate and thereby further determine the exact concentration of peptide standards has been suggested [37]. It is worth mentioning that the initial amounts of the QPrESTs™ are all determined by a similar method, i.e., normalization towards a well-defined His-tagged standard.

In the present study sera from six different cats was analyzed which makes it difficult to draw conclusions about the clinical use. Nevertheless, the cat with acromegaly had, as expected, higher IGF-I than the healthy cats. In healthy humans IGF-II concentrations are higher than IGF-I [38] and this was also observed in the non-acromegalic cats in the present study. In the acromegalic cat IGF-II was not elevated compared to healthy cats while the remaining three proteins showed very high concentrations. In humans, IGF-II is less GH dependent than IGF-I and concentrations in acromegalic people have shown to be both increased and decreased [39,40]. The regulation of growth-related proteins in cats is yet to be explored and this may be facilitated by using MS whereby many components of the IGF-system can be identified. We foresee that the presented mass spectrometry-based method would be a valuable tool for better understanding of this complex system. Using PRM, more proteins can easily be added to the list of proteins to be quantified if suitable peptides for quantification are identified.

## Supporting Information

S1 Table Shotgun screening of three fractions of cat serum obtained by size exclusion chromatography. The results from analysis of Fraction I, II and III are summarized in Tables S1a, b and c, respectively. The four target proteins are high-lighted in green and data on the matching peptides are shown. The Tables were obtained in Proteome Discoverer 1.4 using a standard format.
(PDF)

S1 File Calculations of parameters to determine linear range, limit of detection and limit of quantification. The first sheet shows raw data, area under curve (AUC), of all fragments applied to record calibration curves. The following four sheets show statistical evaluation of IGF-I, IGF-II, IGFBP-3 and IGFBP-5, respectively. The SkyLine software was applied to extract AUC of the fragments and the built in tool Data Analysis in Microsoft Excel 2010 was used to perform linear regression and statistical evaluation.
(XLSX)

S2 File Calculations of concentrations in seven different cat sera. The peak areas of all fragments used for quantification are shown and protein concentrations for each target protein are calculated.
(XLSX)

S3 File Analysis of a human control sample. The control sample was prepared by two different analysts, and analyzed in duplicate experiments. All individual measurements resulted in values well within the specified range of 396–596 ng/mL.
(XLSX)
