# Supplementary material for: Quantitative and Selective Analysis of Feline Growth Related Proteins Using Parallel Reaction Monitoring High Resolution Mass Spectrometry
Source: PLoS One. 2016 Dec 1;11(12):e0167138. doi: 10.1371/journal.pone.0167138 (PMC5132254; doi:10.1371/journal.pone.0167138)
Supplement: S1 Table — The results from analysis of Fraction I, II and III are summarized in Tables S1a, b and c, respectively. The four target proteins are high-lighted in green and data on the matching peptides are shown. The Tables were obtained in Proteome Discoverer 1.4 using a standard format. (PDF) [file pone.0167138.s001.pdf]

Table S1a

Accession	Description	Score	Coverage	# Proteins	# Unique Peptides	# Peptides	# PSMs	# AAs	MW [kDa]	calc. pI						
P49064	Serum albumin OS=Felis catus GN=ALB PE=1 SV=1 - [ALBU_FELCA]	1798,89	88,98	1	6	80	629	608	68,6	5,66						
M3WFW6	Serum albumin OS=Felis catus GN=ALB PE=4 SV=1 - [M3WFW6_FELCA]	1793,67	87,97	1	6	80	624	607	68,5	5,66						
M3WBQ5	Uncharacterized protein OS=Felis catus GN=TF PE=4 SV=1 - [M3WBQ5_FELCA]	273,63	70,20	1	55	56	104	708	78,6	6,95						
M3W2Z1	Uncharacterized protein (Fragment) OS=Felis catus GN=GC PE=4 SV=1 - [M3W2Z1_FELCA]	236,67	78,87	1	35	35	87	478	53,2	5,47						
M3WSI8	Uncharacterized protein OS=Felis catus GN=F2 PE=4 SV=1 - [M3WSI8_FELCA]	169,34	39,07	1	20	20	55	622	70,3	6,21						
M3WEV9	Uncharacterized protein OS=Felis catus GN=TTR PE=4 SV=1 - [M3WEV9_FELCA]	166,35	76,71	1	10	10	49	146	15,5	5,76						
M3WPG6	Uncharacterized protein OS=Felis catus GN=APOA1 PE=4 SV=1 - [M3WPG6_FELCA]	127,07	63,20	1	29	29	50	269	30,6	6,44						
M3W4S3	Uncharacterized protein (Fragment) OS=Felis catus GN=HP PE=4 SV=1 - [M3W4S3_FELCA]	104,89	52,60	2	25	25	46	346	38,3	5,82						
M3X3T9	Uncharacterized protein (Fragment) OS=Felis catus GN=PLG PE=4 SV=1 - [M3X3T9_FELCA]	88,44	41,78	1	21	21	28	821	91,7	7,27						
P07405	Hemoglobin subunit alpha OS=Felis catus GN=HBA PE=1 SV=1 - [HBA_FELCA]	75,90	71,63	1	8	8	27	141	15,3	7,91						
M3WH75	Uncharacterized protein OS=Felis catus GN=APOH PE=4 SV=1 - [M3WH75_FELCA]	62,85	40,87	1	10	10	19	345	38,1	7,97						
M3W0W4	Uncharacterized protein OS=Felis catus GN=A1BG PE=4 SV=1 - [M3W0W4_FELCA]	61,38	27,69	1	7	7	23	502	53,9	5,40						
M3WN87	Uncharacterized protein OS=Felis catus GN=APOA2 PE=4 SV=1 - [M3WN87_FELCA]	61,15	61,00	1	8	8	20	100	11,2	8,10						
M3WMA9	Uncharacterized protein OS=Felis catus GN=A2M PE=4 SV=1 - [M3WMA9_FELCA]	59,39	14,79	1	16	16	22	1481	164,3	6,74						
M3WN61	Uncharacterized protein OS=Felis catus GN=ITIH4 PE=4 SV=1 - [M3WN61_FELCA]	59,23	21,26	1	13	13	20	955	105,5	6,44						
M3W955	Uncharacterized protein OS=Felis catus GN=APOA4 PE=4 SV=1 - [M3W955_FELCA]	55,53	49,87	1	17	17	25	377	42,7	5,81						
M3WPJ9	Uncharacterized protein (Fragment) OS=Felis catus PE=4 SV=1 - [M3WPJ9_FELCA]	53,47	12,69	1	13	13	16	1750	192,5	7,03						
M3WB06	Uncharacterized protein OS=Felis catus GN=FN1 PE=4 SV=1 - [M3WB06_FELCA]	52,82	5,05	1	9	9	13	2475	271,9	5,77						
M3W1H0	Uncharacterized protein (Fragment) OS=Felis catus PE=4 SV=1 - [M3W1H0_FELCA]	48,99	94,34	1	3	8	20	106	11,3	7,08						
M3XFW7	Uncharacterized protein OS=Felis catus GN=CLEC3B PE=4 SV=1 - [M3XFW7_FELCA]	48,83	65,35	1	11	11	14	202	22,3	5,64						
M3W2J4	Uncharacterized protein OS=Felis catus GN=HPX PE=4 SV=1 - [M3W2J4_FELCA]	48,82	34,33	1	11	11	21	466	51,7	7,03						
M3WNF1	Uncharacterized protein OS=Felis catus GN=PON1 PE=4 SV=1 - [M3WNF1_FELCA]	48,42	34,27	1	8	8	14	356	40,1	5,52						
M3WPZ3	Uncharacterized protein (Fragment) OS=Felis catus GN=LOC101090806 PE=4 SV=1 - [M3WPZ3_FELCA]	46,65	84,40	1	2	7	19	109	11,7	7,05						
M3WLL8	Uncharacterized protein OS=Felis catus GN=SERPINC1 PE=4 SV=1 - [M3WLL8_FELCA]	46,49	31,25	1	13	13	19	464	52,4	5,86						
M3W3D2	Uncharacterized protein (Fragment) OS=Felis catus GN=LOC101088220 PE=4 SV=1 - [M3W3D2_FELCA]	41,73	7,38	1	10	10	23	1532	172,2	6,55						
M3VU72	Uncharacterized protein OS=Felis catus GN=AMBP PE=4 SV=1 - [M3VU72_FELCA]	40,84	21,02	1	6	6	11	352	39,1	5,88						
M3X260	Uncharacterized protein (Fragment) OS=Felis catus GN=LOC101087599 PE=4 SV=1 - [M3X260_FELCA]	40,07	18,07	1	9	10	16	703	77,6	6,35						
M3WI95	Uncharacterized protein OS=Felis catus GN=C9 PE=4 SV=1 - [M3WI95_FELCA]	37,50	13,22	1	10	10	13	552	62,0	5,60						
M3WG97	Uncharacterized protein OS=Felis catus GN=AHSG PE=4 SV=1 - [M3WG97_FELCA]	37,33	40,50	1	9	9	16	363	38,8	5,30						
M3WJN1	Uncharacterized protein OS=Felis catus GN=KNG1 PE=4 SV=1 - [M3WJN1_FELCA]	37,23	22,63	1	11	11	15	654	72,9	6,49						
M3WVW8	Uncharacterized protein OS=Felis catus GN=TIMP2 PE=4 SV=1 - [M3WVW8_FELCA]	36,08	43,44	1	8	8	15	221	24,5	8,07						
M3XDZ9	Uncharacterized protein OS=Felis catus GN=IGF1 PE=4 SV=1 - [M3XDZ9_FELCA]	35,76	90,00	1	4	4	10	70	7,6	7,61						
	A2	Sequence	# PSMs	# Proteins	# Protein Groups	Protein Group Accessions	Modifications	ΔCn	q-Value	PEP	XCorr	Charge	MH+ [Da]	ΔM [ppm]	RT [min]	# Missed Cleavages
	High	GPETLcGAELVDALQFVcGDR	5	1	1	M3XDZ9	C6(Carbamidomethyl); C18(Carb	0,0000	0	0,000003843	4,88	2	2307,08159	3,07	63,41	0
	High	RAPQTGIVDEccFR	2	1	1	M3XDZ9	C11(Carbamidomethyl); C12(Car	0,0000	0	0,0000034	3,41	3	1708,78672	-1,61	44,32	1
	High	GFYFNKPTGYGSSSR	2	1	1	M3XDZ9		0,0000	0	0,0003167	2,91	2	1667,78349	1,32	45,82	0
	High	RLEMYcAPLKPAK	1	1	1	M3XDZ9	C6(Carbamidomethyl)	0,0000	0	0,006361	2,56	3	1576,82773	-3,91	43,20	1
M3WCX1	Uncharacterized protein OS=Felis catus GN=SERPINA1 PE=4 SV=1 - [M3WCX1_FELCA]	35,40	27,08	1	9	9	14	421	46,9	6,01						
M3WAN3	Uncharacterized protein (Fragment) OS=Felis catus GN=LOC101085428 PE=4 SV=1 - [M3WAN3_FELCA]	33,90	40,36	1	7	7	11	166	18,8	7,56						
M3X833	Uncharacterized protein (Fragment) OS=Felis catus PE=4 SV=1 - [M3X833_FELCA]	31,78	37,10	1	7	7	12	345	37,3	6,30						
M3X1A4	Uncharacterized protein (Fragment) OS=Felis catus PE=4 SV=1 - [M3X1A4_FELCA]	31,04	20,87	1	3	6	16	369	40,6	7,94						
M3WNX1	Uncharacterized protein OS=Felis catus GN=APOC2 PE=4 SV=1 - [M3WNX1_FELCA]	31,00	61,39	1	8	8	13	101	11,1	5,11						
Q5MGS7	Beta-2-microglobulin OS=Felis catus GN=B2M PE=3 SV=1 - [B2MG_FELCA]	29,75	11,02	1	2	2	8	118	13,7	6,52						
M3WKP2	Uncharacterized protein OS=Felis catus GN=CLU PE=4 SV=1 - [M3WKP2_FELCA]	29,67	13,65	1	6	6	10	447	51,8	5,62						
M3W2B8	Uncharacterized protein OS=Felis catus GN=CFB PE=4 SV=1 - [M3W2B8_FELCA]	29,61	7,18	1	6	6	10	1268	141,0	7,46						
M3WA90	Uncharacterized protein OS=Felis catus GN=CAMP PE=4 SV=1 - [M3WA90_FELCA]	29,03	45,61	1	8	8	11	171	19,4	7,74						
M3WND9	Uncharacterized protein OS=Felis catus GN=LOC101091307 PE=4 SV=1 - [M3WND9_FELCA]	26,38	67,35	1	7	7	8	147	16,1	7,62						
M3WJK3	Uncharacterized protein (Fragment) OS=Felis catus GN=C4BPA PE=4 SV=1 - [M3WJK3_FELCA]	24,50	14,33	1	5	6	8	614	68,7	7,34						
M3WLH3	Uncharacterized protein OS=Felis catus GN=C6 PE=4 SV=1 - [M3WLH3_FELCA]	24,49	7,17	1	4	4	6	935	105,3	7,11						
M3WSC8	Uncharacterized protein OS=Felis catus GN=APOC3 PE=4 SV=1 - [M3WSC8_FELCA]	23,23	33,00	1	4	4	8	100	11,0	8,57						
M3X9P4	Uncharacterized protein (Fragment) OS=Felis catus PE=4 SV=1 - [M3X9P4_FELCA]	23,11	19,12	1	3	6	13	434	47,2	6,37						
M3X8P7	Uncharacterized protein OS=Felis catus GN=APOD PE=4 SV=1 - [M3X8P7_FELCA]	22,46	27,83	1	5	5	7	212	24,0	5,08						
M3W922	Uncharacterized protein (Fragment) OS=Felis catus GN=F5 PE=4 SV=1 - [M3W922_FELCA]	19,90	4,14	1	8	8	9	2102	237,6	6,37						
M3X2U0	Uncharacterized protein OS=Felis catus PE=4 SV=1 - [M3X2U0_FELCA]	19,29	29,70	1	2	3	5	202	22,4	5,39						
D5MTH1	Cystatin C OS=Felis catus GN=CST3 PE=2 SV=1 - [D5MTH1_FELCA]	19,25	42,86	1	3	3	8	147	16,1	9,25						
M3XBM6	Uncharacterized protein (Fragment) OS=Felis catus GN=IGHM PE=4 SV=1 - [M3XBM6_FELCA]	19,14	25,20	1	9	9	11	500	54,8	5,12						
M3W828	Uncharacterized protein OS=Felis catus GN=APOM PE=4 SV=1 - [M3W828_FELCA]	18,72	50,53	1	7	7	7	188	21,2	6,74						
M3W022	Fibrinogen alpha chain OS=Felis catus GN=FGA PE=4 SV=1 - [M3W022_FELCA]	17,56	7,16	1	4	4	8	866	93,6	5,90						
M3W158	Uncharacterized protein (Fragment) OS=Felis catus GN=C8A PE=4 SV=1 - [M3W158_FELCA]	17,28	5,84	1	4	4	6	565	63,4	6,54						
M3WHS6	Uncharacterized protein OS=Felis catus GN=GPX3 PE=4 SV=1 - [M3WHS6_FELCA]	15,33	25,44	1	5	5	6	228	25,4	7,69						
M3WG98	Uncharacterized protein (Fragment) OS=Felis catus GN=FETUB PE=4 SV=1 - [M3WG98_FELCA]	14,02	12,41	1	4	4	5	395	43,3	6,62						
M3WUQ6	Uncharacterized protein OS=Felis catus GN=MST1 PE=4 SV=1 - [M3WUQ6_FELCA]	11,82	8,00	1	4	4	4	725	82,1	7,90						
M3VU75	Uncharacterized protein OS=Felis catus GN=AGP PE=4 SV=1 - [M3VU75_FELCA]	11,72	26,37	1	3	3	4	201	23,1	4,87						
M3XEQ8	Uncharacterized protein (Fragment) OS=Felis catus PE=4 SV=1 - [M3XEQ8_FELCA]	11,47	26,79	1	2	2	3	112	11,9	7,97						
M3X3K7	Uncharacterized protein (Fragment) OS=Felis catus GN=IGFBP3 PE=4 SV=1 - [M3X3K7_FELCA]	10,82	28,66	1	5	5	5	157	18,0	8,92						
	A2	Sequence	# PSMs	# Proteins	# Protein Groups	Protein Group Accessions	Modifications	ΔCn	q-Value	PEP	XCorr	Charge	MH+ [Da]	ΔM [ppm]	RT [min]	# Missed Cleavages
	High	YGQPLPGFDAR	1	1	1	M3X3K7		0,0000	0	0,0001585	2,90	2	1220,61089	4,08	48,49	0
	High	GFcWcVDK	1	1	1	M3X3K7	C3(Carbamidomethyl); C5(Carba	0,0000	0,001	0,04653	2,07	2	1071,44048	1,66	49,95	0
	High	EVEDTLNR	1	1	1	M3X3K7		0,0000	0,002	0,06731	1,67	2	975,47374	-0,48	37,55	0
	High	FLDTLSPR	1	1	1	M3X3K7		0,0000	0,007	0,284	2,50	2	948,51457	-0,40	47,13	0
	Medium	GIHIPNcDRK	1	1	1	M3X3K7	C7(Carbamidomethyl)	0,0000	0,013	0,3515	1,68	2	1209,61577	0,01	37,67	1
M3WQ08	Uncharacterized protein OS=Felis catus GN=IGFBP5 PE=4 SV=1 - [M3WQ08_FELCA]	10,21	13,65	1	3	3	4	271	30,3	8,18						
	A2	Sequence	# PSMs	# Proteins	# Protein Groups	Protein Group Accessions	Modifications	ΔCn	q-Value	PEP	XCorr	Charge	MH+ [Da]	ΔM [ppm]	RT [min]	# Missed Cleavages
	High	QDEEKPLHALLHGR	2	1	1	M3WQ08		0,0000	0	0,0001614	3,47	3	1642,86106	-3,04	43,43	0
	High	ALSMcPPSPLGcELVK	1	1	1	M3WQ08	C5(Carbamidomethyl); C12(Carb	0,0000	0	0,002489	2,32	2	1758,86614	4,17	53,67	0
	High	GVcLNEK	1	1	1	M3WQ08	C3(Carbamidomethyl)	0,0000	0,001	0,03816	1,79	2	819,40087	-2,55	35,93	0
M3W8B6	Uncharacterized protein (Fragment) OS=Felis catus GN=LOC101081299 PE=4 SV=1 - [M3W8B6_FELCA]	9,37	15,77	1	3	3	4	222	25,1	8,16						
M3WKX0	Uncharacterized protein OS=Felis catus GN=LECT2 PE=4 SV=1 - [M3WKX0_FELCA]	9,35	37,75	1	2	2	3	151	16,5	9,00						
M3VZ41	Uncharacterized protein OS=Felis catus GN=TREML1 PE=4 SV=1 - [M3VZ41_FELCA]	8,79	12,06	1	2	2	4	315	33,0	5,22						
M3XG21	Uncharacterized protein OS=Felis catus PE=4 SV=1 - [M3XG21_FELCA]	8,69	15,83	1	4	4	4	240	26,5	7,77						
M3X452	Uncharacterized protein OS=Felis catus GN=LOC101095728 PE=4 SV=1 - [M3X452_FELCA]	8,19	26,89	1	2	2	2	119	13,6	5,00						
M3WJX8	Uncharacterized protein OS=Felis catus GN=CDH1 PE=4 SV=1 - [M3WJX8_FELCA]	7,84	3,41	1	3	3	3	881	97,8	4,77						
M3X5K6	Uncharacterized protein OS=Felis catus PE=4 SV=1 - [M3X5K6_FELCA]	6,52	25,00	2	2	2	2	116	12,4	7,34						
M3WEG4	Uncharacterized protein OS=Felis catus GN=GPLD1 PE=4 SV=1 - [M3WEG4_FELCA]	6,14	5,87	1	3	3	3	835	91,7	6,77						
M3VU02	Uncharacterized protein (Fragment) OS=Felis catus GN=MASP2 PE=4 SV=1 - [M3VU02_FELCA]	6,07	3,47	1	2	2	2	691	76,2	5,97						
M3VZY7	Uncharacterized protein OS=Felis catus GN=CP PE=4 SV=1 - [M3VZY7_FELCA]	5,96	2,47	1	2	2	2	1093	125,1	5,80						
M3WUW9	Uncharacterized protein (Fragment) OS=Felis catus PE=4 SV=1 - [M3WUW9_FELCA]	5,26	2,75	1	2	2	2	801	89,9	6,54						
M3WXH9	Uncharacterized protein OS=Felis catus PE=4 SV=1 - [M3WXH9_FELCA]	4,84	5,21	2	2	2	2	384	42,3	8,53						
M3WWL4	Uncharacterized protein OS=Felis catus PE=4 SV=1 - [M3WWL4_FELCA]	2,95	2,35	1	2	2	2	1487	166,6	6,90						
M3WYA8	Uncharacterized protein OS=Felis catus PE=4 SV=1 - [M3WYA8_FELCA]	0,00	10,33	1	2	2	2	213	24,4	5,49						

Table S1b

Accession	Description	Score	Coverage	# Proteins	# Unique Peptides	# Peptides	# PSMs	# AAs	MW [kDa]	calc. pI						
M3WFW6	Serum albumin OS=Felis catus GN=ALB PE=4 SV=1 - [M3WFW6_FELCA]	675,90	81,38	1	6	60	319	607	68,5	5,66						
P49064	Serum albumin OS=Felis catus GN=ALB PE=1 SV=1 - [ALBU_FELCA]	653,93	82,40	1	6	60	313	608	68,6	5,66						
M3WBQ5	Uncharacterized protein OS=Felis catus GN=TF PE=4 SV=1 - [M3WBQ5_FELCA]	162,40	52,40	1	34	34	67	708	78,6	6,95						
M3WPJ9	Uncharacterized protein (Fragment) OS=Felis catus PE=4 SV=1 - [M3WPJ9_FELCA]	159,26	8,06	1	12	12	48	1750	192,5	7,03						
M3W2Z1	Uncharacterized protein (Fragment) OS=Felis catus GN=GC PE=4 SV=1 - [M3W2Z1_FELCA]	123,89	60,25	1	22	22	43	478	53,2	5,47						
M3WPG6	Uncharacterized protein OS=Felis catus GN=APOA1 PE=4 SV=1 - [M3WPG6_FELCA]	86,51	63,20	1	27	27	39	269	30,6	6,44						
M3WEV9	Uncharacterized protein OS=Felis catus GN=TTR PE=4 SV=1 - [M3WEV9_FELCA]	85,07	71,92	1	8	8	27	146	15,5	5,76						
M3WSI8	Uncharacterized protein OS=Felis catus GN=F2 PE=4 SV=1 - [M3WSI8_FELCA]	80,36	24,92	1	12	12	29	622	70,3	6,21						
M3W4S3	Uncharacterized protein (Fragment) OS=Felis catus GN=HP PE=4 SV=1 - [M3W4S3_FELCA]	77,90	46,24	2	19	19	34	346	38,3	5,82						
M3WN87	Uncharacterized protein OS=Felis catus GN=APOA2 PE=4 SV=1 - [M3WN87_FELCA]	67,75	66,00	1	9	9	25	100	11,2	8,10						
M3W828	Uncharacterized protein OS=Felis catus GN=APOM PE=4 SV=1 - [M3W828_FELCA]	58,75	88,30	1	14	14	23	188	21,2	6,74						
M3WND9	Uncharacterized protein OS=Felis catus GN=LOC101091307 PE=4 SV=1 - [M3WND9_FELCA]	48,05	63,27	1	1	10	18	147	16,1	7,62						
P07412	Hemoglobin subunit beta-A/B OS=Felis catus GN=HBB PE=1 SV=1 - [HBB_FELCA]	39,82	69,18	1	2	11	17	146	15,9	7,24						
M3WNX1	Uncharacterized protein OS=Felis catus GN=APOC2 PE=4 SV=1 - [M3WNX1_FELCA]	36,14	61,39	1	7	7	13	101	11,1	5,11						
M3WG97	Uncharacterized protein OS=Felis catus GN=AHSG PE=4 SV=1 - [M3WG97_FELCA]	30,58	40,50	1	8	8	12	363	38,8	5,30						
M3W3D2	Uncharacterized protein (Fragment) OS=Felis catus GN=LOC101088220 PE=4 SV=1 - [M3W3D2_FELCA]	29,16	3,39	1	5	5	11	1532	172,2	6,55						
M3VX72	Uncharacterized protein (Fragment) OS=Felis catus PE=4 SV=1 - [M3VX72_FELCA]	28,33	32,67	1	2	2	9	101	11,0	9,36						
M3X3T9	Uncharacterized protein (Fragment) OS=Felis catus GN=PLG PE=4 SV=1 - [M3X3T9_FELCA]	22,66	13,15	1	8	8	9	821	91,7	7,27						
M3XDZ9	Uncharacterized protein OS=Felis catus GN=IGF1 PE=4 SV=1 - [M3XDZ9_FELCA]	22,59	71,43	1	3	3	7	70	7,6	7,61						
	A2	Sequence	# PSMs	# Proteins	# Protein Groups	Protein Group Accessions	Modifications	ΔCn	q-Value	PEP	XCorr	Charge	MH+ [Da]	ΔM [ppm]	RT [min]	# Missed Cleavages
	High	RAPQTGIVDEccFR	1	1	1	M3XDZ9	C11(Carbamidomethyl); C12(Car	0,0000	0	4,098E-07	4,00	3	1708,79312	2,14	44,38	1
	High	GPETLcGAELVDALQFVcGDR	3	1	1	M3XDZ9	C6(Carbamidomethyl); C18(Carb	0,0000	0	0,0001081	3,97	2	2307,07964	2,22	63,76	0
	High	GFYFNKPTGYGSSSR	3	1	1	M3XDZ9		0,0000	0	0,00006442	3,12	2	1667,78447	1,91	46,32	0
M3WSC8	Uncharacterized protein OS=Felis catus GN=APOC3 PE=4 SV=1 - [M3WSC8_FELCA]	21,95	33,00	1	5	5	9	100	11,0	8,57						
M3WUW9	Uncharacterized protein (Fragment) OS=Felis catus PE=4 SV=1 - [M3WUW9_FELCA]	21,45	6,62	1	5	5	8	801	89,9	6,54						
M3X9F2	Uncharacterized protein OS=Felis catus GN=LOC101082240 PE=4 SV=1 - [M3X9F2_FELCA]	19,78	33,11	1	4	4	7	151	17,3	9,20						
M3VUG6	Uncharacterized protein OS=Felis catus GN=KRT1 PE=4 SV=1 - [M3VUG6_FELCA]	19,50	6,67	1	5	5	7	630	64,7	7,47						
M3W2J4	Uncharacterized protein OS=Felis catus GN=HPX PE=4 SV=1 - [M3W2J4_FELCA]	19,10	23,82	1	6	6	12	466	51,7	7,03						
M3WH75	Uncharacterized protein OS=Felis catus GN=APOH PE=4 SV=1 - [M3WH75_FELCA]	18,61	20,00	1	4	4	6	345	38,1	7,97						
M3WT57	Uncharacterized protein (Fragment) OS=Felis catus GN=PPBP PE=4 SV=1 - [M3WT57_FELCA]	18,59	41,13	1	4	4	7	124	13,1	8,94						
M3WN61	Uncharacterized protein OS=Felis catus GN=ITIH4 PE=4 SV=1 - [M3WN61_FELCA]	17,89	10,58	1	6	6	7	955	105,5	6,44						
M3WHS6	Uncharacterized protein OS=Felis catus GN=GPX3 PE=4 SV=1 - [M3WHS6_FELCA]	17,30	21,93	1	4	4	7	228	25,4	7,69						
M3W0W4	Uncharacterized protein OS=Felis catus GN=A1BG PE=4 SV=1 - [M3W0W4_FELCA]	16,87	13,55	1	3	3	6	502	53,9	5,40						
M3WCX1	Uncharacterized protein OS=Felis catus GN=SERPINA1 PE=4 SV=1 - [M3WCX1_FELCA]	16,82	16,39	1	6	6	8	421	46,9	6,01						
M3WKP2	Uncharacterized protein OS=Felis catus GN=CLU PE=4 SV=1 - [M3WKP2_FELCA]	15,19	11,41	1	4	4	6	447	51,8	5,62						
M3X6T9	Uncharacterized protein OS=Felis catus GN=PSMB1 PE=4 SV=1 - [M3X6T9_FELCA]	14,91	17,43	1	3	3	5	241	26,4	7,39						
M3X9P4	Uncharacterized protein (Fragment) OS=Felis catus PE=4 SV=1 - [M3X9P4_FELCA]	14,78	13,59	1	2	4	7	434	47,2	6,37						
P07405	Hemoglobin subunit alpha OS=Felis catus GN=HBA PE=1 SV=1 - [HBA_FELCA]	14,49	36,88	1	4	4	6	141	15,3	7,91						
M3W955	Uncharacterized protein OS=Felis catus GN=APOA4 PE=4 SV=1 - [M3W955_FELCA]	13,03	23,34	1	7	7	7	377	42,7	5,81						
M3X1A4	Uncharacterized protein (Fragment) OS=Felis catus PE=4 SV=1 - [M3X1A4_FELCA]	12,02	14,91	1	2	4	7	369	40,6	7,94						
M3XBM6	Uncharacterized protein (Fragment) OS=Felis catus GN=IGHM PE=4 SV=1 - [M3XBM6_FELCA]	11,69	11,40	1	3	3	5	500	54,8	5,12						
M3XE51	Uncharacterized protein OS=Felis catus GN=IGF2 PE=4 SV=1 - [M3XE51_FELCA]	9,68	49,25	1	2	2	3	67	7,5	6,44						
	A2	Sequence	# PSMs	# Proteins	# Protein Groups	Protein Group Accessions	Modifications	ΔCn	q-Value	PEP	XCorr	Charge	MH+ [Da]	ΔM [ppm]	RT [min]	# Missed Cleavages
	High	AYRPSETLcGGELVDTLQFVcGDR	2	1	1	M3XE51	C9(Carbamidomethyl); C21(Carb	0,0000	0	0,00006455	3,97	3	2743,28647	1,79	57,58	0
	High	GIVEEccFR	1	1	1	M3XE51	C6(Carbamidomethyl); C7(Carba	0,0000	0	0,003194	2,84	2	1169,50408	-3,23	44,74	0
M3WPZ3	Uncharacterized protein (Fragment) OS=Felis catus GN=LOC101090806 PE=4 SV=1 - [M3WPZ3_FELCA]	9,26	45,87	1	4	4	4	109	11,7	7,05						
M3W022	Fibrinogen alpha chain OS=Felis catus GN=FGA PE=4 SV=1 - [M3W022_FELCA]	9,04	3,12	1	2	2	3	866	93,6	5,90						
M3WJN1	Uncharacterized protein OS=Felis catus GN=KNG1 PE=4 SV=1 - [M3WJN1_FELCA]	8,94	6,12	1	4	4	5	654	72,9	6,49						
M3X0G8	Amyloid protein A OS=Felis catus GN=SAA1 PE=4 SV=1 - [M3X0G8_FELCA]	8,48	20,16	1	2	2	3	129	14,3	8,16						
D5MTH1	Cystatin C OS=Felis catus GN=CST3 PE=2 SV=1 - [D5MTH1_FELCA]	7,23	32,65	1	2	2	8	147	16,1	9,25						
M3WMA9	Uncharacterized protein OS=Felis catus GN=A2M PE=4 SV=1 - [M3WMA9_FELCA]	6,36	5,74	1	6	6	7	1481	164,3	6,74						
M3WI95	Uncharacterized protein OS=Felis catus GN=C9 PE=4 SV=1 - [M3WI95_FELCA]	6,16	6,88	1	3	3	3	552	62,0	5,60						
M3X8P7	Uncharacterized protein OS=Felis catus GN=APOD PE=4 SV=1 - [M3X8P7_FELCA]	4,84	8,96	1	2	2	4	212	24,0	5,08						
M3VV58	Uncharacterized protein OS=Felis catus GN=C5 PE=4 SV=1 - [M3VV58_FELCA]	3,92	2,21	1	3	3	3	1678	188,8	6,62						
M3VXR1	Uncharacterized protein OS=Felis catus GN=KRT10 PE=4 SV=1 - [M3VXR1_FELCA]	3,28	4,68	1	2	2	2	556	58,3	5,38						
M3W922	Uncharacterized protein (Fragment) OS=Felis catus GN=F5 PE=4 SV=1 - [M3W922_FELCA]	0,00	3,04	1	3	3	3	2102	237,6	6,37						
M3W5B5	Uncharacterized protein (Fragment) OS=Felis catus GN=LRP2 PE=4 SV=1 - [M3W5B5_FELCA]	0,00	1,71	1	2	2	3	4626	518,9	5,12						

Table S1c

Accession	Description	Score	Coverage	# Proteins	# Unique Peptides	# Peptides	# PSMs	# AAs	MW [kDa]	calc. pI						
M3WFW6	Serum albumin OS=Felis catus GN=ALB PE=4 SV=1 - [M3WFW6_FELCA]	299,38	67,22	1	3	34	107	607	68,5	5,66						
P49064	Serum albumin OS=Felis catus GN=ALB PE=1 SV=1 - [ALBU_FELCA]	287,98	68,26	1	3	34	107	608	68,6	5,66						
M3XDZ9	Uncharacterized protein OS=Felis catus GN=IGF1 PE=4 SV=1 - [M3XDZ9_FELCA]	65,26	90,00	1	5	5	20	70	7,6	7,61						
	A2	Sequence	# PSMs	# Proteins	# Protein Groups	Protein Group Accessions	Modifications	ΔCn	q-Value	PEP	XCorr	Charge	MH+ [Da]	ΔM [ppm]	RT [min]	# Missed Cleavages
	High	GPETLcGAELVDALQFVcGDR	6	1	1	M3XDZ9	C6(Carbamidomethyl); C18(Carb	0,0000	0	0,07446	5,41	2	2307,07842	1,69	63,64	0
	High	RAPQTGIVDEccFR	10	1	1	M3XDZ9	C11(Carbamidomethyl); C12(Car	0,0000	0	0,03537	4,01	3	1708,79404	2,68	44,62	1
	High	GFYFNKPTGYGSSSR	2	1	1	M3XDZ9		0,0000	0	0,2173	3,20	3	1667,77842	-1,72	46,36	0
	High	RLEMYcAPLKPAK	1	1	1	M3XDZ9	C6(Carbamidomethyl)	0,0000	0	0,1666	3,12	3	1576,82865	-3,33	43,07	1
	High	APQTGIVDEccFR	1	1	1	M3XDZ9	C10(Carbamidomethyl); C11(Car	0,0000	0	0,04087	3,03	2	1552,69011	1,13	47,05	0
M3W2Z1	Uncharacterized protein (Fragment) OS=Felis catus GN=GC PE=4 SV=1 - [M3W2Z1_FELCA]	30,73	18,20	1	5	5	9	478	53,2	5,47						
P14469	Fibrinogen beta chain (Fragment) OS=Felis catus GN=FGB PE=1 SV=1 - [FIBB_FELCA]	29,60	100,00	1	2	2	10	20	2,3	4,01						
M3WPG6	Uncharacterized protein OS=Felis catus GN=APOA1 PE=4 SV=1 - [M3WPG6_FELCA]	28,90	36,06	1	8	8	10	269	30,6	6,44						
M3WEV9	Uncharacterized protein OS=Felis catus GN=TTR PE=4 SV=1 - [M3WEV9_FELCA]	27,05	50,68	1	4	4	6	146	15,5	5,76						
M3W828	Uncharacterized protein OS=Felis catus GN=APOM PE=4 SV=1 - [M3W828_FELCA]	25,01	56,38	1	5	5	7	188	21,2	6,74						
M3WPJ9	Uncharacterized protein (Fragment) OS=Felis catus PE=4 SV=1 - [M3WPJ9_FELCA]	19,44	3,49	1	4	4	6	1750	192,5	7,03						
M3W4S3	Uncharacterized protein (Fragment) OS=Felis catus GN=HP PE=4 SV=1 - [M3W4S3_FELCA]	17,15	18,50	2	6	6	7	346	38,3	5,82						
M3XE51	Uncharacterized protein OS=Felis catus GN=IGF2 PE=4 SV=1 - [M3XE51_FELCA]	17,13	49,25	1	2	2	4	67	7,5	6,44						
	A2	Sequence	# PSMs	# Proteins	# Protein Groups	Protein Group Accessions	Modifications	ΔCn	q-Value	PEP	XCorr	Charge	MH+ [Da]	ΔM [ppm]	RT [min]	# Missed Cleavages
	High	AYRPSETLcGGELVDTLQFVcGDR	3	1	1	M3XE51	C9(Carbamidomethyl); C21(Carb	0,0000	0	0,0855	5,16	2	2743,27471	-2,49	57,62	0
	High	GIVEEccFR	1	1	1	M3XE51	C6(Carbamidomethyl); C7(Carba	0,0000	0,004	0,3774	2,91	2	1169,49944	-7,19	45,60	0
M3XEQ8	Uncharacterized protein (Fragment) OS=Felis catus PE=4 SV=1 - [M3XEQ8_FELCA]	15,55	26,79	1	2	2	4	112	11,9	7,97						
M3W3D2	Uncharacterized protein (Fragment) OS=Felis catus GN=LOC101088220 PE=4 SV=1 - [M3W3D2_FELCA]	14,48	1,37	1	3	3	5	1532	172,2	6,55						
M3WN87	Uncharacterized protein OS=Felis catus GN=APOA2 PE=4 SV=1 - [M3WN87_FELCA]	11,72	27,00	1	3	3	4	100	11,2	8,10						
M3WNX1	Uncharacterized protein OS=Felis catus GN=APOC2 PE=4 SV=1 - [M3WNX1_FELCA]	11,17	18,81	1	2	2	4	101	11,1	5,11						
M3WBQ5	Uncharacterized protein OS=Felis catus GN=TF PE=4 SV=1 - [M3WBQ5_FELCA]	10,15	7,34	1	5	5	6	708	78,6	6,95						
M3WPZ3	Uncharacterized protein (Fragment) OS=Felis catus GN=LOC101090806 PE=4 SV=1 - [M3WPZ3_FELCA]	10,01	42,20	1	3	3	4	109	11,7	7,05						
M3WSI8	Uncharacterized protein OS=Felis catus GN=F2 PE=4 SV=1 - [M3WSI8_FELCA]	7,45	4,82	1	2	2	3	622	70,3	6,21						
M3WMA9	Uncharacterized protein OS=Felis catus GN=A2M PE=4 SV=1 - [M3WMA9_FELCA]	7,37	1,35	1	2	2	3	1481	164,3	6,74						
M3WSC8	Uncharacterized protein OS=Felis catus GN=APOC3 PE=4 SV=1 - [M3WSC8_FELCA]	7,10	28,00	1	3	3	3	100	11,0	8,57						
M3WG97	Uncharacterized protein OS=Felis catus GN=AHSG PE=4 SV=1 - [M3WG97_FELCA]	6,87	12,67	1	2	2	2	363	38,8	5,30						
M3WGY4	Uncharacterized protein OS=Felis catus GN=KRT14 PE=4 SV=1 - [M3WGY4_FELCA]	6,71	8,02	1	3	3	3	474	51,7	5,16						
M3XBM6	Uncharacterized protein (Fragment) OS=Felis catus GN=IGHM PE=4 SV=1 - [M3XBM6_FELCA]	6,24	7,20	1	2	2	3	500	54,8	5,12						
M3VUG6	Uncharacterized protein OS=Felis catus GN=KRT1 PE=4 SV=1 - [M3VUG6_FELCA]	5,97	5,40	1	3	3	3	630	64,7	7,47						
P07412	Hemoglobin subunit beta-A/B OS=Felis catus GN=HBB PE=1 SV=1 - [HBB_FELCA]	5,47	15,75	2	2	2	2	146	15,9	7,24						
M3WJN1	Uncharacterized protein OS=Felis catus GN=KNG1 PE=4 SV=1 - [M3WJN1_FELCA]	5,13	3,21	1	2	2	2	654	72,9	6,49						
M3W955	Uncharacterized protein OS=Felis catus GN=APOA4 PE=4 SV=1 - [M3W955_FELCA]	4,72	4,77	1	2	2	2	377	42,7	5,81						
